# *In-situ* and real-time growth observation of high-quality protein crystals under quasi-microgravity on earth

**DOI:** 10.1038/srep22127

**Published:** 2016-02-26

**Authors:** Akira Nakamura, Jun Ohtsuka, Tatsuki Kashiwagi, Nobutaka Numoto, Noriyuki Hirota, Takahiro Ode, Hidehiko Okada, Koji Nagata, Motosuke Kiyohara, Ei-ichiro Suzuki, Akiko Kita, Hitoshi Wada, Masaru Tanokura

**Affiliations:** 1 Department of Applied Biological Chemistry, Graduate School of Agricultural and Life Sciences, The University of Tokyo, 1-1-1 Yayoi, Bunkyo-ku, Tokyo 113-8657, Japan; 2 Institute for Innovation, Ajinomoto Co. Inc., 1-1 Suzuki-cho, Kawasaki-ku, Kawasaki, Kanagawa 210-8681, Japan; 3 Research Reactor Institute, Kyoto University, Kumatori, Sennan, Osaka 590-0494, Japan; 4 National Institute for Materials Science, 3-13 Sakura, Tsukuba, Ibaraki 305-0003, Japan; 5 Kiyohara Optics Inc., 6-23-2 Shinjuku, Shinjuku-ku, Tokyo 160-0022, Japan

## Abstract

Precise protein structure determination provides significant information on life science research, although high-quality crystals are not easily obtained. We developed a system for producing high-quality protein crystals with high throughput. Using this system, gravity-controlled crystallization are made possible by a magnetic microgravity environment. In addition, *in-situ* and real-time observation and time-lapse imaging of crystal growth are feasible for over 200 solution samples independently. In this paper, we also report results of crystallization experiments for two protein samples. Crystals grown in the system exhibited magnetic orientation and showed higher and more homogeneous quality compared with the control crystals. The structural analysis reveals that making use of the magnetic microgravity during the crystallization process helps us to build a well-refined protein structure model, which has no significant structural differences with a control structure. Therefore, the system contributes to improvement in efficiency of structural analysis for “difficult” proteins, such as membrane proteins and supermolecular complexes.

X-ray crystallography is a powerful and broadly applicable tool for determining the three-dimensional structures of protein molecules. Once we obtain structural information at atomic resolution, we can consider the relationship between protein structures and functions in detail, which is crucial to the design of new drugs, engineering of practical enzymes, and development of novel biotechnology. However, it is often difficult to obtain high-quality protein crystals that diffract X-rays to high angles and enable precise structure determination. Thus, there is a great demand for technological innovation in protein crystallization.

The microgravity environment attained in the International Space Station has been utilized to produce high-quality protein crystals, since the density convection due to gravitational force, which disturbs the ideal crystallization process, is eliminated in space^1^. On the other hand, Wakayama *et al*. demonstrated that a quasi-microgravity environment achieved by magnetic forces can also improve the quality of diamagnetic protein crystals^2^. The magnetic force in the *z* direction is defined as the product of the magnetic field, ***B***_***z***_, and magnetic field gradient, d***B***_***z***_/d*z*, and can compensate for gravitational forces acting on diamagnetic substances, when applied vertically and upwards. A magnetic force-based quasi-microgravity (magnetic quasi-microgravity) environment has been utilized to obtain protein crystals with improved qualities^3^,^4^,^5^. The influence of magnetic force on the flow of a diamagnetic aqueous solution in the magnetic microgravity environment has recently been estimated by numerical calculations^6^. We here report the details of a protein crystallization system recently developed, and the results of crystallization experiments performed using the developed system, demonstrating the advantage of the system in protein crystallography.

## Results

### Features of the system developed

**Figure 1:**
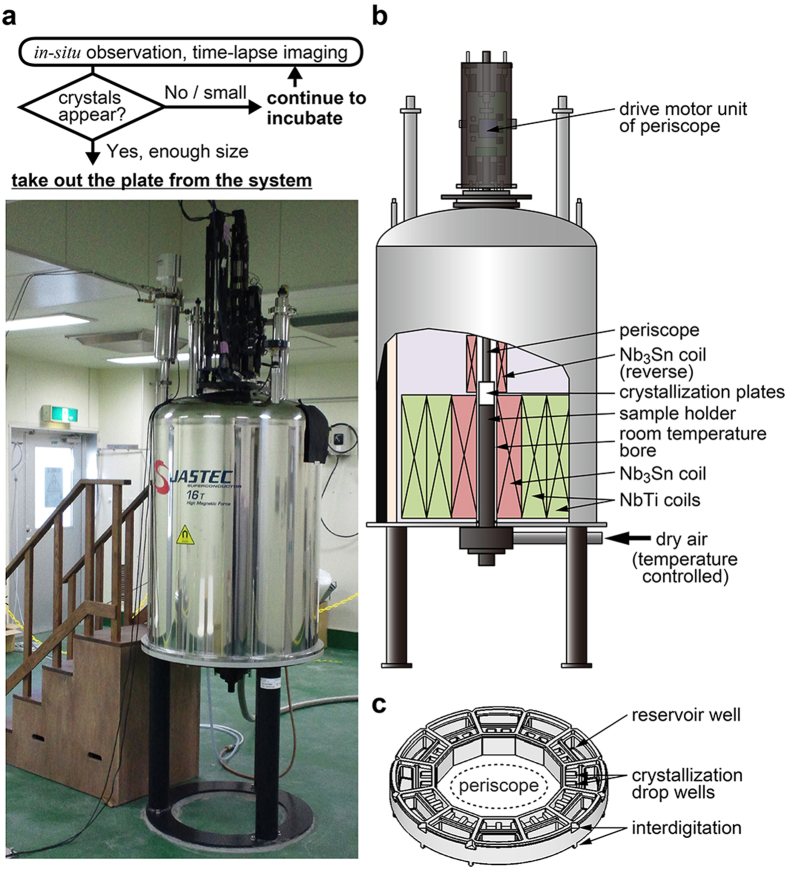
Overview of the developed system. (**a**) Outward appearance of the system. (**b**) Schematic illustration of the system. (**c**) Schematic illustration of a crystallization plate.

Using the magneto-scientific technology, we have developed an integrated system for high-throughput and high-quality protein crystal formation (Fig. 1). This system is composed of a superconducting magnet, an inverted periscope, and other equipment suited for both producing high-quality protein crystals and *in situ* observation of crystal growth under the magnetic quasi-microgravity conditions. The superconducting magnet has a specific configuration of two component coils wound in opposite directions and designed to generate a magnetic field with a steep gradient around the crystallization samples (Fig. 1b), causing a strong upward magnetic force that can cancel out the gravitational force acting on a water droplet (water levitation conditions)^7^. In other words, the system realizes a quasi-microgravity environment on earth. The periscope is made mainly from feeble magnetic materials and is designed to record the crystal growth visually in the environment with a strong magnetic field and magnetic force. The other units, such as the temperature controlling unit and the crystallization plate (Fig. 1c), are designed to assist in the crystallization experiments, the observation of crystal growth, and the handling of the system.

Crystallization experiments with this system can be performed at a constant temperature between 4 °C and 20 °C (±0.1 °C) for more than one month. Custom-made 12-well crystallization plates are used for the protein crystallization (Fig. 1c). Each of the crystallization wells has one reservoir well with a maximum volume of 60 μl and two crystallization drop wells with maximum volumes of 5 μl. Projections at the bottom of the plates fit to depressions at the top of the plates, and the interdigitating structures contribute to the vertical alignment of the well position as well as the stabilized stack of the plates. Ten crystallization plates are stacked in the room-temperature bore of the superconducting magnet where *B*_*z*_d*B*_*z*_/d*z* = −1,160 ~ −1,510 T^2^/m, which correspond to *z* = 45 ~ 100 mm from the center of the Nb_3_Sn main coil and effective gravity of 0.15 ~ −0.11 G for water droplet.

The periscope can be remote controlled for vertical movement, horizontal rotation, and focus adjustment. A snapshot of the crystallization drop well can be taken from the inner circumferential side-wall of the toroidal plate (Fig. 1c). All the crystallization drop wells can be sequentially observed *in situ* without rotating the plates and without removing the plates from the system. Since the positional shift of the crystallization plates causes a change of the magnetic force environment and, thus, causes a change in the effective gravity for the crystallization drop, this *in-situ* observation device enables us to see whether crystals are formed without inducing a disturbance in the crystallization drops. Furthermore, using this system, we can check the status of the crystal growth at any time. Through the real-time observation, we can determine when the crystals have grown sufficiently and thus the plate should be taken out from the system, and we can evaluate whether the crystallization conditions are adequate as early as possible. Time-lapse imaging is another useful function of the system. Once users schedule the capture order of the wells, the system automatically and repeatedly takes photographs of the crystallization drop wells. The results will then provide us with a variety of information on the protein crystal growth, including when the crystals form, the sites from which they emerge, how they grow, and whether or not they exhibit a magnetic orientation.

### *In situ* observation of crystal growth under magnetic quasi-microgravity conditions

**Figure 2:**
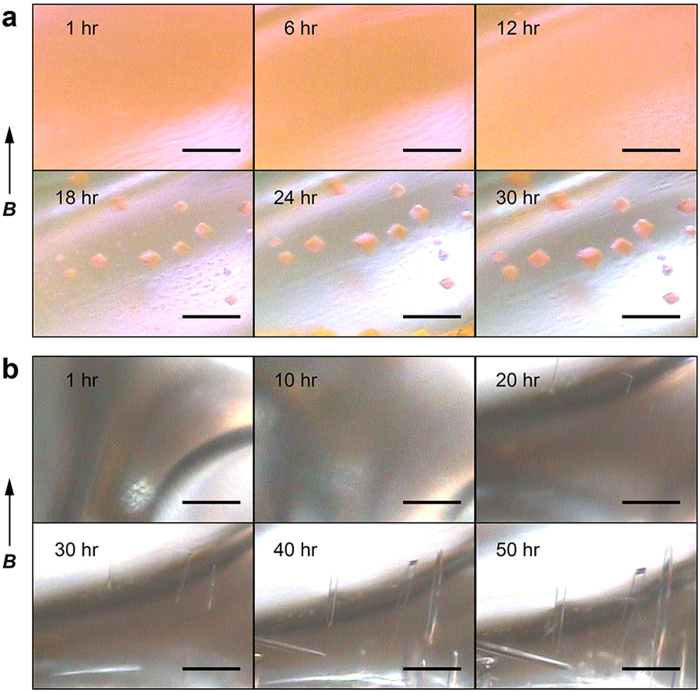
Time-lapse snapshots of protein crystal growth in the magnetic microgravity. (**a**) Crystal growth of a GFP-like fluorescent protein, monomeric Kusabira-Orange (mKO). (**b**) Crystal growth of a zinc-metalloprotease, ZPαβ. Elapsed time from the crystallization setup is indicated at the top-left of each image. The directions of the magnetic fields during the crystallization experiments are indicated by arrows with the letter ***B***. Scale bars, 200 μm.

**Figure 3:**
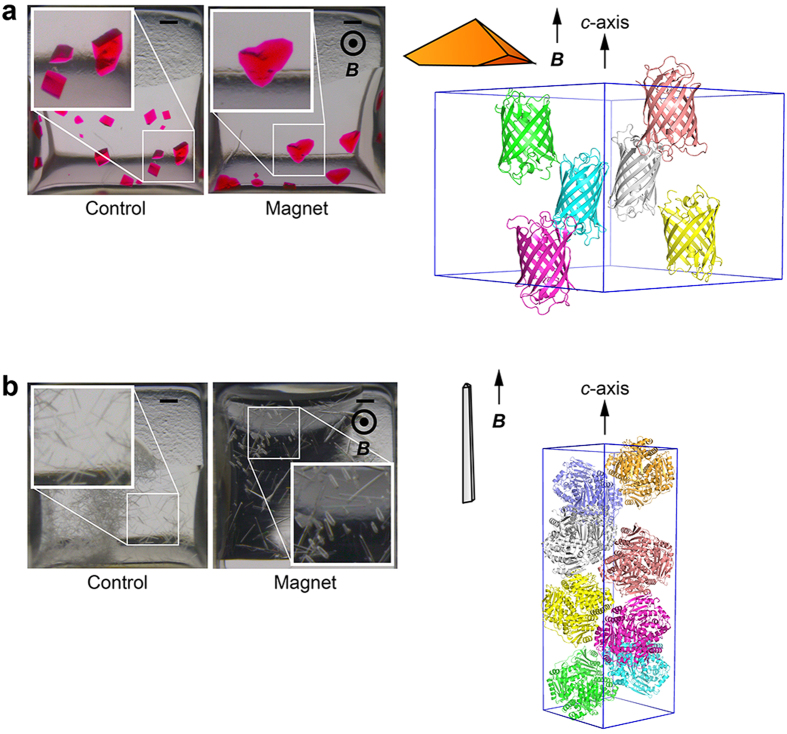
Protein crystals and their magnetic orientation. Crystals of (**a**) mKO and (**b**) ZPαβ grown in the control experiments (Control) and using the system developed (Magnet) are shown. The left two pictures are the results of the crystallization experiments (top view). Insets show enlarged views of the boxed regions. Scale bars, 200 μm. The magnetic field direction is perpendicular to the display. The right panels indicate cartoons of obtained crystals and crystal packings of the protein molecules. The figures of protein molecules were prepared with PyMOL (The PyMOL Molecular Graphics System, Version 1.5.0.3. Schrödinger, LLC. http://www.pymol.org).

In this article, we show results of case studies with the system developed using two protein samples: a GFP-like fluorescent protein, monomeric Kusabira-Orange from *Fungia concinna* (mKO), and a heterodimeric zinc protease from *Thermus thermophilus* HB8 (ZPαβ). The protein crystallization experiments were carried out and the crystal growth was recorded in a series of photographs (every 10 min). The time-lapse pictures indicated that mKO and ZPαβ crystals were grown in a day and two days, respectively (Fig. 2 and Supplementary Movies 1 and 2), under the conditions described in the Methods section. The movies also show that the drops were shrunk and the menisci of the solutions were lowered according to the progress of the vapor diffusion. In the case of mKO, the crystals appeared simultaneously at various positions in the drop, as soon as the orange color of the solution became clear in about 12 hours after the crystallization setup (Fig. 2a and Supplementary Movie 1). The vertex of the triangular pyramid-shaped mKO crystals was aligned to the direction of the magnetic fields, showing the magnetic orientation. The X-ray diffraction images indicated that the *c*-axis of the trigonal mKO crystal is oriented parallel to the magnetic field direction (Fig. 3a). It has been thought that magnetic orientation is attributable to the diamagnetic anisotropy of protein molecules, especially the anisotropy derived from planar peptide bonds and aromatic rings of amino acid residues^8^. Therefore, the pleat of a β-sheet as well as the helical axis of an α-helix becomes parallel to the magnetic field direction. As the mKO protein adopts a β-barrel structure and the peptide planes of the β-barrel are almost parallel to the *c*-axis, the mKO crystals exhibit a *c*-axis magnetic orientation. Long pillar-shaped ZPαβ crystals also exhibited the *c*-axis magnetic orientation (Fig. 3b).

### Comparisons of crystal size, quality, and structure

**Figure 4:**
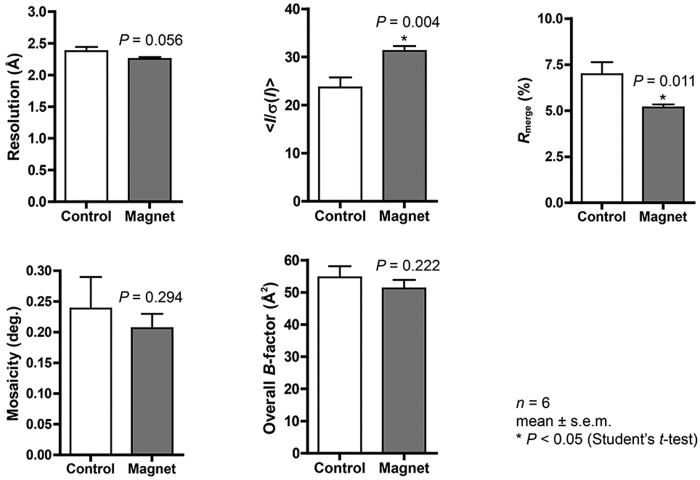
X-ray diffraction data comparisons for mKO crystals. Maximum resolution, overall <*I*/σ(*I*)>, overall *R*_merge_, crystal mosaicity, and overall *B*-factor values are compared between the data from the crystals obtained in the control experiments (Control), and those obtained using the system developed (Magnet) (Supplementary Table 1). The one-tailed Student’s *t*-tests for 6 datasets were carried out. Error bars, standard error of the mean. The overall <*I*/σ(*I*)> and overall *R*_merge_ values are significantly improved compared with the control (*P < 0.05).

**Figure 5:**
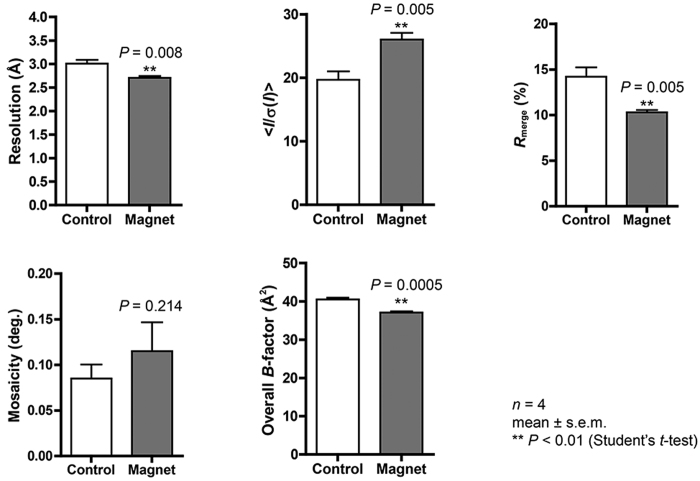
X-ray diffraction data comparisons for ZPαβ crystals. Maximum resolution, overall <*I*/σ(*I*)>, overall *R*_merge_, crystal mosaicity, and overall *B*-factor values are compared between the data from the crystals obtained in the control experiments (Control), and those obtained using the system developed (Magnet) (Supplementary Table 2). The one-tailed Student’s *t*-tests for 4 datasets were carried out. Error bars, standard error of the mean. The maximum resolution, overall <*I*/σ(*I*)>, overall *R*_merge_, and overall *B*-factor values are significantly improved compared with the control (**P < 0.01).

The comparison of mKO crystals grown under the magnetic quasi-microgravity conditions (240 ± 130 μm for the longest side of crystals; mean ± s.d.) with those grown without the application of a magnetic field (200 ± 50 μm) revealed no significant differences in crystal sizes. On the other hand, the ZPαβ crystals obtained using the system are significantly wider than the control crystals (25 ± 4 μm vs 16 ± 3 μm for the shortest side of crystals). Crystal quality was evaluated based on the results of the X-ray diffraction experiments and the following statistical analyses (Supplementary Tables 1 and 2, Figs 4 and 5). The crystal quality of ZPαβ, which involves maximum resolution, signal-to-noise ratio (<*I*/σ(*I*)>), *R*_merge_ value, and overall *B*-factor, is significantly improved when the crystal was grown in the system (Student’s *t*-test: *P* < 0.01). It seems that the larger volume of magnetically oriented ZPαβ crystals grown in the magnetic quasi-microgravity environment contributes to the enhancement of these parameters. As for the mKO crystals, overall <*I*/σ(*I*)> and *R*_merge_ values are significantly improved over those of the control crystals. The improvement in the maximum resolution approaches acceptable levels of statistical significance (*P* = 0.056). The crystal mosaicity and overall *B*-factor value show some marginal but not significant improvements (Supplementary Table 1 and Fig. 4). These facts suggest that the quality of the mKO crystals is improved by using the magnetic quasi-microgravity environment. In addition, a comparison of the standard errors of the data imply that the qualities of the crystals obtained in the present system are more homogeneous than those of the control crystals (Supplementary Tables 1 and 2).

The crystal structures of mKO and ZPαβ were determined using each of the X-ray diffraction intensity dataset collected in this study and the refined structures were analyzed (Supplementary Tables 1 and 2). In regard to the mKO structures, the crystallographic *R*_work_/*R*_free_ factors for the six ‘control’ and six ‘magnet’ datasets are 0.219 ± 0.012/0.249 ± 0.010 and 0.204 ± 0.004/0.232 ± 0.005, respectively (mean ± s.e.m.). The result indicates that the structures of the ‘magnet’ crystals are well-refined compared with those of the ‘control’ crystals mainly because of the higher resolution limit. On the other hand, structural comparisons of all possible combinations of the twelve structures, containing both the ‘magnet’ and ‘control’ structures, reveals that these structures are almost identical with r.m.s. deviations of 0.10 ± 0.02 Å for 213 Cα atoms and 0.49 ± 0.08 Å for 1711 atoms including side chains of amino acid residues (mean ± s.d.). Saijo *et al*. reported that a homogeneous and static magnetic field of 10 T improved perfection of hen egg-white lysozyme crystals with reduced *B*-factors and without structural changes from the 0 T crystal control^9^. Although our experimental environment supplies a strong magnetic force in addition to the high magnetic field, there is no significant differences between the structures of crystals grown within the gradient magnetic field and those grown without the application of magnetic field, either. The structural analysis of ZPαβ also shows a similar result to the case of mKO. The crystallographic *R*_work_/*R*_free_ factors for the four ‘control’ and four ‘magnet’ datasets of ZPαβ are 0.204 ± 0.005/0.256 ± 0.006 and 0.187 ± 0.006/0.243 ± 0.006, respectively (mean ± s.e.m.). All of the ZPαβ structures are quite similar one another with r.m.s.d. values of 0.26 ± 0.05 Å for 808 Cα atoms and 0.80 ± 0.12 Å for 6307 atoms (mean ± s.d.). These results suggest that the magnetic quasi-microgravity induces no structural changes in protein molecules, while the environment contributes to improvement quality of protein crystals and increasing crystal size in some cases. In addition, magnetic orientation shown in this study may not affect a local structure of amino acid residues but certainly help ideal growth of crystals.

## Discussion

There are many reports describing approaches to improve quality of protein crystals, especially the resolution limit, and the methods can be categorized into three groups. One is to change properties of a protein of interest by amino-acid substitution (e.g. the surface entropy reduction^10^), chemical modifications (e.g. methylation^11^), etc. Another is to change crystallization fields using a microgravity environment in space^1^, hydrogel^12^, magnetic fields^13^, etc. The other is called post-crystallization treatments, such as dehydration, crystal annealing^14^, and so on. Our method is belong to the second group, which makes use of the reduced natural convection and high magnetic fields. We think our method is one of the easiest in the second group, because it is not necessary to have a special skill when setting up a crystallization experiment.

The benefit of the application of a magnetic force during protein crystallization has been known to date^3^,^5^,^15^. In this study, we evaluated for the first time the quality of crystals grown in the gradient magnetic field by means of the statistical analysis, suggesting that the crystallization under the magnetic quasi-microgravity conditions contributes to not only improvement but also homogenization of crystal quality. This is important because, in the case of crystals with weak diffraction intensity or X-ray-sensitive crystals, it may be necessary to use hundreds of crystals to collect one complete diffraction dataset, and such a task requires a crystallization method for producing homogeneous crystals. We also expect that protein crystallization with the developed system would be useful technology for phasing by means of the traditional multiple isomorphous replacement technique and for protein serial femtosecond crystallography using X-ray free-electron laser radiation with a liquid jet containing microcrystals^16^.

The results of this study suggest that the crystallization system developed has the potential to provide protein crystals of improved quality and may have a great advantage for the precise structure determination and structural analysis of so-called difficult protein samples, such as membrane proteins and supramolecular complexes, which are known to be crystallized with poor quality by conventional methods. Moreover, the real-time observation of the system should be useful for a wide range of studies, such as for determining the kinetics of the crystallization process of various substances in the magnetic microgravity environment.

## Methods

### Sample preparation

mKO protein was expressed in *Escherichia coli* and purified using a protocol similar to that used for the homologous fluorescent protein, monomeric Azami-Green^17^. Each subunit of the ZPαβ protein from *Thermus thermophilus* HB8 was co-expressed in *E. coli* Rosetta (DE3) (Novagen). The cells harboring ZPαβ encoding genes on pET-11 (α subunit) and pET-28 (β subunit) were cultured in LB containing ampicillin, kanamycin, and chloramphenicol at 37 °C until OD600 = 0.6. Protein expression was induced by the addition of 0.5 mM IPTG, and then the cells were cultured at 25 °C overnight and harvested. The collected cells were resuspended with 20 mM Tris-HCl (pH 8.0) and 50 mM NaCl, and disrupted by sonication. The cell debris was removed by centrifuging at 40,000 g for 30 minutes, and the lysate was incubated at 80 °C for 30 minutes to denature proteins from the expression host. After centrifugation, the supernatant was loaded onto a Zn-IMAC column (Ni^2+^ of Ni-NTA (QIAGEN) was replaced by Zn^2+^). The column was washed with 20 mM Tris-HCl (pH 8.0) and 5 mM imidazole, and the bound proteins were eluted with 20 mM Tris-HCl (pH 8.0) and 20 mM imidazole. The eluate was dialyzed against 20 mM Tris-HCl (pH 8.0) and loaded onto a RESOURCE Q column (GE Healthcare) equilibrated with 20 mM Tris-HCl (pH 8.0). His-tagged ZPαβ protein was eluted with a linear gradient of 0 to 0.5 M NaCl. ZPαβ was further purified by a gel-filtration column, Superdex 200 HR 10/30 (GE Healthcare), equilibrated with 20 mM Tris-HCl (pH 8.0) and 150 mM NaCl, and concentrated to 10 mg/ml for crystallization.

### Crystallization

Protein crystallization was performed with a custom-made plate (Fig. 1c) using the sitting-drop vapor-diffusion method. Crystallization drops were made by mixing 1 μl of 10 mg/ml protein solution and an equal volume of precipitant solution in the crystallization drop wells, and the drops were equilibrated against 20 μl of the precipitant solution in the reservoir well. The precipitant solution for mKO was comprised of 0.1 M CHES-NaOH (pH 9.7) and 40–50% *v/v* PEG-600. The precipitant solution for ZPαβ was comprised of 1 M sodium-potassium phosphate (pH 6.1–7.1). After the crystallization plates were sealed, the plates were set in the room-temperature bore of the superconducting magnet and incubated at 20 °C for 2 weeks. The magnetic field strength (***B***_***z***_), the production of the field strength and field gradient (***B***_***z***_d***B***_***z***_/d***z***), and the effective gravity for a water droplet in the crystallization environment were 8.4 ~ 14.0 T, −1,160 ~ −1,510 T^2^/m, and −0.11 ~ 0.15 G including 0 G, respectively. The control experiment was carried out in a typical incubator under the same conditions as described above, except for the magnetic field.

### Time-lapse imaging

The coordinates of the drops targeted for imaging were registered to the scheduling program and the capture cycle was set to be 10 minutes. Time-lapse imaging was carried out from 1 to 50 hours after the crystallization plates were set up in the room-temperature bore of the superconducting magnet.

### X-ray diffraction experiments

The mKO crystals were frozen directly in a stream of nitrogen gas at 95 K. The ZPαβ crystals were soaked in a cryo-protectant solution containing 25% *v/v* ethylene glycol in the precipitant solution for 5 sec before the 95 K flash cooling. X-ray diffraction experiments were performed using synchrotron radiation at beamlines NE-3A and NW-12A of PF-AR (Tsukuba, Japan) and Cu-Kα X-rays generated by an in-house apparatus (FR-E SuperBright; RIGAKU, Tokyo, Japan). The diffraction intensity datasets were collected as a total of 360 frames with an oscillation angle of 0.5°, and an exposure time of 0.5 sec (PF-AR) or 60 sec (Lab) per frame.

### Evaluation of crystal quality

X-ray diffraction data were indexed, integrated, and scaled using the programs XDS^18^,^19^ and SCALA^19^ of the CCP4 software suite^20^. A criterion of *R*_merge_ ≤ 40% was used for determination of the maximum resolution cutoff value. Overall *B*-factor values were calculated using SFCHECK^21^ by the Wilson plot and by the Patterson origin peak for the mKO and ZPαβ datasets, respectively, since some of the ZPαβ datasets (Control-1 and Control-2) showed a low resolution limit worse than 3 Å (Supplementary Tables 1 and 2). The maximum resolution, overall <*I*/σ(*I*)>, overall *R*_merge_, crystal mosaicity, and overall *B*-factor values from the control crystals and the crystals grown in the system developed were compared using the one-tailed Student’s *t*-test (Figs 4 and 5). The crystal structures of mKO and ZPαβ were determined by the molecular replacement method using the previously reported mKO structure (PDB code: 2ZMU) and the β subunit structure of ZPαβ (PDB code: 3EOQ), respectively, as a search model with the program PHASER^22^ on PHENIX^23^. Several cycles of model building and refinement were carried out using the program COOT^24^ and PHENIX. Structural comparison was performed with the program LSQKAB^25^ of CCP4.

## Additional Information

**How to cite this article**: Nakamura, A. *et al*. *In-situ* and real-time growth observation of high-quality protein crystals under quasi-microgravity on earth. *Sci. Rep*. **6**, 22127; doi: 10.1038/srep22127 (2016).

## Supplementary Material

Supplementary Information

Supplementary Movie S1

Supplementary Movie S2
