# Supplementary material for: In-situ and real-time growth observation of high-quality protein crystals under quasi-microgravity on earth
Source: Sci Rep. 2016 Feb 26;6:22127. doi: 10.1038/srep22127 (PMC4768258; doi:10.1038/srep22127)
Supplement: Supplementary Information [file srep22127-s1.doc]

Supplementary Information for

***In-situ* and real-time growth observation of high-quality protein crystals under quasi-microgravity on earth**

Akira Nakamura^1^, Jun Ohtsuka^1^, Tatsuki Kashiwagi^2^, Nobutaka Numoto^3,†^, Noriyuki Hirota^4^, Takahiro Ode^5^, Hidehiko Okada^4^, Koji Nagata^1^, Motosuke Kiyohara^5^, Ei-ichiro Suzuki^2^, Akiko Kita^3^, Hitoshi Wada^4^, and Masaru Tanokura^1,*^

^1^Department of Applied Biological Chemistry, Graduate School of Agricultural and Life Sciences, The University of Tokyo, 1-1-1 Yayoi, Bunkyo-ku, Tokyo 113-8657, Japan

^2^Institute for Innovation, Ajinomoto, Co. Inc., 1-1 Suzuki-cho, Kawasaki-ku, Kawasaki, Kanagawa 210-8681, Japan

^3^Research Reactor Institute, Kyoto University, Kumatori, Sennan, Osaka 590-0494, Japan

^4^National Institute for Materials Science, 3-13 Sakura, Tsukuba, Ibaraki 305-0003, Japan

^5^Kiyohara Optics, Inc., 6-23-2 Shinjuku, Shinjuku-ku, Tokyo 160-0022, Japan

List:

Supplementary Table 1: X-ray diffraction intensity data collection and refinement statistics for mKO crystals.

Supplementary Table 2: X-ray diffraction intensity data collection and refinement statistics for ZPαβ crystals.

Supplementary Movie 1: Time-lapse movie of mKO crystal growth in the magnetic quasi-microgravity environment.

Supplementary Movie 2: Time-lapse movie of ZPαβ crystal growth in the magnetic quasi-microgravity environment.

^*^Corresponding author: Prof. Dr. Masaru Tanokura

^†^Present address:

Medical Research Institute, Tokyo Medical and Dental University, 1-5-45 Yushima, Bunkyo-ku, Tokyo 113-8510, Japan

**Supplementary Table 1. X-ray diffraction intensity data collection and refinement statistics for mKO crystals**

| **Data collection** | **Control-1** | **Control-2** | **Control-3** | **Control-4** | **Control-5** | **Control-6** | **mean±s.e.m.^§^** |
| --- | --- | --- | --- | --- | --- | --- | --- |
| **X-ray source** | Lab | Lab | Lab | Lab | PF-AR NE3A | PF-AR NE3A | - |
| **Wavelength (Å)** | 1.5418 | 1.5418 | 1.5418 | 1.5418 | 1.0000 | 1.0000 | - |
| **Resolution range (Å)^*^** | 45.06–2.39  (2.52–2.39) | 44.89–2.61  (2.75–2.61) | 44.95–2.19  (2.30–2.19) | 44.96–2.26  (2.39–2.26) | 44.89–2.50  (2.64–2.50) | 44.88–2.31  (2.43–2.31) | 2.38 ± 0.06 |
| **Redundancy** | 10.7 | 9.4 | 10.3 | 10.7 | 11.1 | 11.1 | - |
| **Completeness (%)^*^** | 100.0 (100.0) | 100.0 (100.0) | 99.9 (99.7) | 100.0 (100.0) | 100.0 (100.0) | 100.0 (100.0) | - |
| **<*I*/σ(*I*)>^*^** | 15.6 (4.8) | 23.8 (4.8) | 22.1 (4.7) | 22.5 (5.7) | 28.3 (6.2) | 29.7 (6.2) | 23.7 ± 2.1 |
| ***R*_merge_ (%)^*^** | 9.8 (40.0) | 7.0 (39.9) | 6.9 (40.0) | 7.2 (39.6) | 5.9 (40.0) | 5.1 (40.0) | 7.0 ± 0.7 |
| **Crystal mosaicity (°)** | 0.39 | 0.40 | 0.21 | 0.18 | 0.11 | 0.14 | 0.24 ± 0.05 |
| **Overall *B*-factor (Å^2^)^‡^** | 51.4 | 63.4 | 45.0 | 45.7 | 63.9 | 58.5 | 54.6 ± 3.5 |
| **Space group** | *P*3_1_21 | *P*3_1_21 | *P*3_1_21 | *P*3_1_21 | *P*3_1_21 | *P*3_1_21 | - |
| **Cell parameters *a*, *c* (Å)** | 98.7, 90.1 | 97.9, 89.8 | 98.7, 89.9 | 98.7, 89.9 | 98.4, 89.8 | 98.4, 89.8 | - |
| **Refinement** | **Control-1** | **Control-2** | **Control-3** | **Control-4** | **Control-5** | **Control-6** | **mean±s.e.m.^§^** |
| **Resolution range (Å)** | 43.27–2.39 | 42.40–2.61 | 39.79-2.19 | 39.79-2.26 | 39.72–2.50 | 38.50–2.31 | - |
| ***R*_work_**^¶^  ***R*_free_**^¶^ | 0.279  0.300 | 0.200  0.247 | 0.204  0.239 | 0.210  0.232 | 0.215  0.236 | 0.204  0.241 | 0.219 ± 0.012  0.249 ± 0.010 |
| **No. of atoms** |  |  |  |  |  |  |  |
| **macromolecules** | 1711 | 1711 | 1711 | 1711 | 1711 | 1711 | - |
| **solvent** | 117 | 76 | 223 | 187 | 71 | 117 | - |
| **Average *B* factors (Å^2^)** |  |  |  |  |  |  |  |
| **macromolecule** | 37.2 | 41.7 | 33.7 | 32.0 | 45.9 | 45.1 | 39.3 ± 2.4 |
| **solvent** | 41.7 | 41.8 | 47.9 | 43.5 | 48.8 | 52.4 | 46.0 ± 1.8 |
| **R.m.s. deviations** |  |  |  |  |  |  |  |
| **bond lengths (Å)** | 0.005 | 0.005 | 0.007 | 0.007 | 0.002 | 0.004 | - |
| **bond angles (°)** | 1.119 | 1.206 | 1.283 | 1.271 | 0.968 | 1.127 | - |
| **Ramachandran plot^#^** |  |  |  |  |  |  |  |
| **Favoured (%)** | 97.6 | 98.1 | 98.1 | 98.1 | 98.1 | 98.1 | - |
| **Allowed (%)** | 2.4 | 1.9 | 1.4 | 1.9 | 1.9 | 1.9 | - |
| **Outliers (%)** | 0.0 | 0.0 | 0.5 | 0.0 | 0.0 | 0.0 | - |

| **Data collection** | **Magnet-1** | **Magnet-2** | **Magnet-3** | **Magnet-4** | **Magnet-5** | **Magnet-6** | **mean±s.e.m.^§^** |
| --- | --- | --- | --- | --- | --- | --- | --- |
| **X-ray source** | Lab | Lab | Lab | Lab | PF-AR NE3A | PF-AR NE3A | - |
| **Wavelength (Å)** | 1.5418 | 1.5418 | 1.5418 | 1.5418 | 1.0000 | 1.0000 | - |
| **Resolution range (Å)^*^** | 45.12–2.18  (2.30–2.18) | 44.94–2.23  (2.35–2.23) | 44.98–2.27  (2.40–2.27) | 44.87–2.21  (2.33–2.21) | 49.18–2.39  (2.51–2.39) | 44.85–2.25  (2.37–2.25) | 2.25 ± 0.03 |
| **Redundancy** | 10.4 | 10.5 | 10.7 | 10.5 | 11.1 | 11.0 | - |
| **Completeness (%)^*^** | 99.9 (99.5) | 100.0 (100.0) | 100.0 (100.0) | 100.0 (100.0) | 100.0 (100.0) | 100.0 (100.0) | - |
| **<*I*/σ(*I*)>^*^** | 27.8 (5.0) | 33.3 (5.3) | 30.6 (6.1) | 30.6 (5.2) | 34.6 (6.3) | 30.7 (6.1) | 31.3 ± 1.0 |
| ***R*_merge_ (%)^*^** | 5.6 (40.0) | 5.0 (39.8) | 5.5 (39.9) | 5.4 (40.0) | 4.6 (39.9) | 5.0 (40.0) | 5.2 ± 0.2 |
| **Crystal mosaicity (°)** | 0.29 | 0.25 | 0.17 | 0.22 | 0.17 | 0.14 | 0.21 ± 0.02 |
| **Overall *B*-factor (Å^2^)^‡^** | 45.4 | 48.0 | 49.1 | 47.3 | 62.8 | 54.5 | 51.2 ± 2.6 |
| **Space group** | *P*3_1_21 | *P*3_1_21 | *P*3_1_21 | *P*3_1_21 | *P*3_1_21 | *P*3_1_21 | - |
| **Cell parameters *a*, *c* (Å)** | 98.6, 90.2 | 98.9, 89.9 | 98.4, 90.0 | 98.7, 89.7 | 98.4, 89.7 | 98.6, 89.7 | - |
| **Refinement** | **Magnet-1** | **Magnet-2** | **Magnet-3** | **Magnet-4** | **Magnet-5** | **Magnet-6** | **mean±s.e.m.^§^** |
| **Resolution range (Å)** | 43.26–2.18 | 39.80–2.23 | 39.78–2.27 | 43.23–2.21 | 39.69–2.39 | 33.17–2.25 | - |
| ***R*_work_**^¶^  ***R*_free_**^¶^ | 0.209  0.230 | 0.218  0.249 | 0.189  0.215 | 0.198  0.236 | 0.203  0.236 | 0.207  0.223 | 0.204 ± 0.004  0.232 ± 0.005 |
| **No. of atoms** |  |  |  |  |  |  |  |
| **macromolecules** | 1711 | 1711 | 1711 | 1711 | 1711 | 1711 | - |
| **solvent** | 164 | 152 | 179 | 169 | 100 | 136 | - |
| **Average *B-*factors (Å^2^)** |  |  |  |  |  |  |  |
| **macromolecule** | 33.5 | 36.2 | 34.0 | 35.9 | 47.4 | 42.3 | 38.2 ± 2.2 |
| **solvent** | 46.2 | 46.8 | 45.4 | 45.8 | 53.7 | 50.8 | 48.1 ± 1.4 |
| **R.m.s. deviations** |  |  |  |  |  |  |  |
| **bond lengths (Å)** | 0.006 | 0.007 | 0.012 | 0.008 | 0.004 | 0.004 | - |
| **bond angles (°)** | 1.246 | 1.301 | 1.448 | 1.268 | 1.119 | 1.137 | - |
| **Ramachandran plot^#^** |  |  |  |  |  |  |  |
| **Favoured (%)** | 98.1 | 97.6 | 98.6 | 98.1 | 98.1 | 98.1 | - |
| **Allowed (%)** | 1.9 | 2.4 | 1.4 | 1.4 | 1.9 | 1.9 | - |
| **Outliers (%)** | 0.0 | 0.0 | 0.0 | 0.5 | 0.0 | 0.0 | - |

The prefixes “Control-“ and “Magnet-“ of the dataset name indicate that the X-ray diffraction dataset were collected from a crystal obtained in the control experiment and in the magnetic quasi-microgravity environment, respectively.

^*^ Values for the highest resolution shell are in parentheses.

^‡^ Overall *B*-factor values were calculated using SFCHECK on the basis of the Wilson plot.

^§^ Values for the mean ± the standard error of the mean (s.e.m) were shown for maximum resolution, overall <*I*/σ(*I*)>, overall *R*_merge_, crystal mosaicity, overall *B*-factor, *R*_work_, *R*_free_, and average *B*-factors.

^¶^ *R*_work_ = Σ ||*F*_obs_| – |*F*_calc_|| / Σ |*F*_obs_|. *R*_free_ is the same as *R*_work_, but for 5% subset of all reflections that were never used in crystallographic refinement.

# Calculated using MolProbity.

**Supplementary Table 2. X-ray diffraction intensity data collection and refinement statistics for ZPαβ crystals**

| **Data collection** | **Control-1** | **Control-2** | **Control-3** | **Control-4** | **mean±s.e.m.^§^** |
| --- | --- | --- | --- | --- | --- |
| **X-ray source** | PF-AR NW12A | PF-AR NW12A | PF-AR NE3A | PF-AR NE3A | - |
| **Wavelength (Å)** | 1.0000 | 1.0000 | 1.0000 | 1.0000 | - |
| **Resolution range (Å)^*^** | 48.97–3.21  (3.39–3.21) | 48.98–3.06  (3.23–3.06) | 48.71–2.87  (3.03–2.87) | 49.12–2.89  (3.04–2.89) | 3.01 ± 0.08 |
| **Redundancy** | 14.2 | 14.2 | 14.3 | 14.3 | - |
| **Completeness (%)^*^** | 100.0 (100.0) | 100.0 (100.0) | 100.0 (100.0) | 100.0 (100.0) | - |
| **<*I*/σ(*I*)>^*^** | 18.1 (8.1) | 16.7 (7.5) | 21.5 (7.8) | 22.4 (8.0) | 19.7 ± 1.4 |
| ***R*_merge_ (%)^*^** | 16.4 (39.9) | 15.5 (40.0) | 12.6 (40.0) | 12.3 (39.8) | 14.2 ± 1.0 |
| **Crystal mosaicity (°)** | 0.13 | 0.08 | 0.06 | 0.07 | 0.09 ± 0.02 |
| **Overall *B*-factor (Å^2^)^‡^** | 41.8 | 40.5 | 39.8 | 40.0 | 40.5 ± 0.5 |
| **Space group** | *P*4_3_2_1_2 | *P*4_3_2_1_2 | *P*4_3_2_1_2 | *P*4_3_2_1_2 | - |
| **Cell parameters *a*, *c* (Å)** | 87.0, 243.0 | 86.9, 243.1 | 87.2, 243.6 | 87.3, 243.5 | - |
| **Refinement** | **Control-1** | **Control-2** | **Control-3** | **Control-4** | **mean±s.e.m.^§^** |
| **Resolution range (Å)** | 48.97–3.21 | 48.98–3.06 | 43.62–2.87 | 49.12–2.89 | - |
| ***R*_work_**^¶^  ***R*_free_**^¶^ | 0.192  0.240 | 0.209  0.264 | 0.213  0.267 | 0.201  0.252 | 0.204 ± 0.005  0.256 ± 0.006 |
| **No. of atoms** |  |  |  |  |  |
| **macromolecules** | 6307 | 6307 | 6307 | 6307 | - |
| **phosphate** | 45 | 35 | 5 | 10 | - |
| **solvent** | 0 | 0 | 80 | 102 | - |
| **Average *B*-factors (Å^2^)** |  |  |  |  |  |
| **macromolecule** | 24.5 | 20.3 | 27.6 | 26.4 | 24.7 ± 1.6 |
| **phosphate** | 52.6 | 37.6 | 34.4 | 49.2 | 43.4 ± 4.4 |
| **solvent** | - | - | 24.8 | 22.3 | 23.5 ± 1.3 |
| **R.m.s. deviations** |  |  |  |  |  |
| **bond lengths (Å)** | 0.002 | 0.002 | 0.004 | 0.003 | - |
| **bond angles (°)** | 0.521 | 0.463 | 0.627 | 0.596 | - |
| **Ramachandran plot^#^** |  |  |  |  |  |
| **Favoured (%)** | 97.5 | 97.1 | 97.3 | 97.0 | - |
| **Allowed (%)** | 2.4 | 2.6 | 2.6 | 2.6 | - |
| **Outliers (%)** | 0.1 | 0.3 | 0.1 | 0.4 | - |

| **Data collection** | **Magnet-1** | **Magnet-2** | **Magnet-3** | **Magnet-4** | **mean±s.e.m.^§^** |
| --- | --- | --- | --- | --- | --- |
| **X-ray source** | PF-AR NW12A | PF-AR NW12A | PF-AR NE3A | PF-AR NE3A | - |
| **Wavelength (Å)** | 1.0000 | 1.0000 | 1.0000 | 1.0000 | - |
| **Resolution range (Å)^*^** | 48.92–2.66  (2.80–2.66) | 49.02–2.80  (2.95–2.80) | 49.14–2.62  (2.76–2.62 | 48.71–2.74  (2.89–2.74) | 2.70 ± 0.04 |
| **Redundancy** | 14.4 | 14.3 | 14.4 | 14.4 | - |
| **Completeness (%)^*^** | 100.0 (100.0) | 100.0 (100.0) | 100.0 (100.0) | 99.8 (99.6) | - |
| **<*I*/σ(*I*)>^*^** | 28.7 (8.0) | 23.9 (8.0) | 26.6 (8.1) | 24.9 (8.0) | 26.0 ± 1.1 |
| ***R*_merge_ (%)^*^** | 10.0 (39.9) | 10.8 (39.9) | 9.7 (40.0) | 10.7 (40.0) | 10.3 ± 0.3 |
| **Crystal mosaicity (°)** | 0.21 | 0.09 | 0.08 | 0.08 | 0.12 ± 0.03 |
| **Overall *B*-factor (Å^2^)^‡^** | 36.5 | 38.0 | 36.7 | 37.1 | 37.1 ± 0.3 |
| **Space group** | *P*4_3_2_1_2 | *P*4_3_2_1_2 | *P*4_3_2_1_2 | *P*4_3_2_1_2 | - |
| **Cell parameters *a*, *c* (Å)** | 86.7, 243.5 | 87.3, 241.9 | 87.3, 243.5 | 87.2, 243.6 | - |
| **Refinement** | **Magnet-1** | **Magnet-2** | **Magnet-3** | **Magnet-4** | **mean±s.e.m.^§^** |
| **Resolution range (Å)** | 48.92–2.66 | 49.02–2.80 | 49.14–2.62 | 43.59–2.74 | - |
| ***R*_work_**^¶^  ***R*_free_**^¶^ | 0.174  0.230 | 0.192  0.248 | 0.181  0.239 | 0.202  0.258 | 0.187 ± 0.006  0.243 ± 0.006 |
| **No. of atoms** |  |  |  |  |  |
| **macromolecules** | 6307 | 6307 | 6307 | 6307 | - |
| **phosphate** | 50 | 15 | 30 | 25 | - |
| **solvent** | 235 | 117 | 250 | 167 | - |
| **Average *B* factors (Å^2^)** |  |  |  |  |  |
| **macromolecule** | 30.3 | 31.6 | 31.1 | 30.1 | 30.8 ± 0.4 |
| **phosphate** | 56.5 | 57.6 | 55.7 | 51.5 | 55.3 ± 1.3 |
| **solvent** | 32.7 | 28.5 | 32.8 | 30.4 | 31.1 ± 1.0 |
| **R.m.s. deviations** |  |  |  |  |  |
| **bond lengths (Å)** | 0.004 | 0.003 | 0.005 | 0.005 | - |
| **bond angles (°)** | 0.676 | 0.563 | 0.700 | 0.643 | - |
| **Ramachandran plot^#^** |  |  |  |  |  |
| **Favoured (%)** | 97.8 | 97.1 | 97.9 | 98.0 | - |
| **Allowed (%)** | 2.2 | 2.5 | 2.1 | 1.9 | - |
| **Outliers (%)** | 0.0 | 0.4 | 0.0 | 0.1 | - |

The prefixes “Control-“ and “Magnet-“ of the dataset name indicate that the X-ray diffraction dataset were collected from a crystal obtained in the control experiment and in the magnetic quasi-microgravity environment, respectively.

^*^ Values for the highest resolution shell are in parentheses.

^‡^ Overall *B*-factor values were calculated using SFCHECK on the basis of the Patterson origin peak.

^§^ Values for the mean ± the standard error of the mean (s.e.m.) were shown for maximum resolution, overall <*I*/σ(*I*)>, overall *R*_merge_, crystal mosaicity, and overall *B*-factor, *R*_work_, *R*_free_, and average *B*-factors..

^¶^ *R*_work_ = Σ ||*F*_obs_| – |*F*_calc_|| / Σ |*F*_obs_|. *R*_free_ is the same as *R*_work_, but for 5% subset of all reflections that were never used in crystallographic refinement.

# Calculated using MolProbity.
